# A Real‐Life Assessment of Injectable Polynucleotides High Purification Technology in Aesthetic Medicine for Skin Rejuvenation

**DOI:** 10.1111/jocd.70532

**Published:** 2026-01-02

**Authors:** Lanza Eliana, Perna Anna, Bizzarri Stefania, Santoro Concetta, Cerutti Luisa, Dybala Agnieszka, Brunoro Andrea, Boffi Laura, Prussia Carolina, Saretta Simone

**Affiliations:** ^1^ Estederm Catania Italy; ^2^ Studio Perna Napoli Italy; ^3^ Villa Alba Rome Italy; ^4^ Ini villa alba centro storico Rome Italy; ^5^ Università Sapienza Roma Rome Italy; ^6^ Centro Medico Polispecialistico Analysis Terracina Italy; ^7^ Beauty Formula Milano Milan Italy; ^8^ Bovisio Polimedica Bovisio Masciago Italy; ^9^ Medical Statistics Consultant Locarno Switzerland; ^10^ Medicina Estetica Zazzaron Treviso Italy

**Keywords:** PN HPT, polynucleotides, Polynucleotides High Purification Technology, skin quality, skin rejuvenation

## Abstract

**Background:**

Polynucleotides (PN) are innovative polymers that improve skin hydration and elasticity, serving as alternatives to traditional dermal fillers. Medical devices based on PN High Purification Technology (PN HPT) represent a new generation of injectable products designed to restore hydration, elasticity, and overall tissue quality. PN HPT is known for its clinical versatility and excellent tolerability.

**Aims:**

To assess the real‐world performance and safety of Plinest (40 mg/2 mL of PN HPT in a 2 mL pre‐filled syringe), a CE‐marked class III medical device for skin rejuvenation of the face, neck, and décolleté.

**Methods:**

This observational clinical data collection involved 66 adult patients, each of whom could receive treatment in up to three areas (face, neck, décolleté). Patients underwent three sessions of intradermal PN HPT injections. A total of 106 questionnaires were collected: 47 for the face, 33 for the neck, and 26 for the décolleté. Performance was evaluated using the Global Clinical Improvement Scale (GCI‐S) and Global Aesthetic Improvement Scale (GAIS). Safety was monitored through spontaneous adverse event (AE) reporting.

**Results:**

Clinician‐reported outcomes showed visible improvement in 100% of facial treatments, with 53.5% rated as “marked” or “excellent.” In the neck and décolleté areas, moderate to significant improvements were observed in over 93% and 88% of cases, respectively. Patient satisfaction ranged from 97% to 100%. No serious AEs occurred.

**Conclusions:**

PN HPT demonstrated a favorable tolerability and performance profile in real‐life aesthetic practice, supporting its role in improving skin quality and reducing signs of aging.

## 1 Introduction

Skin aging is a multifactorial biological process influenced by intrinsic factors, such as chronological aging and genetic predisposition, as well as extrinsic factors, including environmental pollution and lifestyle habits [1]. The visible effects of this process—namely, loss of elasticity, increased skin roughness, thinning of the dermis, and the appearance of fine lines—are particularly evident in exposed areas like the face, neck, and décolleté. These regions are not only more vulnerable to physiological aging and environmental damage but also play a crucial role in social perception, making them prime targets for aesthetic interventions [1].

In recent years, aesthetic medicine has increasingly responded to the demand for non‐invasive or minimally invasive procedures aimed at improving skin quality and promoting natural dermal rejuvenation to reduce visible signs of aging [2]. This evolution has gone hand in hand with a shift in interventional strategies, from volumetric correction and wrinkle filling to a more holistic and natural approach to restore the skin's functional integrity. In this context, it has gained considerable attention as a strategy for activating physiological regeneration and repair processes without altering the patient's facial expression or anatomy [3].

Polynucleotides HPT (High Purification Technology) are innovative polymers used in aesthetic fields. These long‐chain DNA fragments, purified and adapted for intradermal use, show viscoelastic properties, allowing temporary filling effects, especially with the increase of extracellular matrix [4]. Unlike synthetic fillers, PN HPT contributes to long‐term dermal renewal by improving skin hydration, elasticity, and tone. Several studies have highlighted their efficacy in treating both chronoaging and photoaging, confirming their safety profiles and clinical versatility [5, 6]. In addition to their use in aesthetic rejuvenation, PN HPT has shown clinically relevant benefits in other dermatological and regenerative contexts. Their safety and effectiveness have been demonstrated in the treatment of striae albae, atrophic acne scars, and as a priming strategy before other aesthetic treatments, such as laser or filler procedures [4, 6, 7].

Plinest [8] is a CE‐marked class III injectable device that contains highly purified polynucleotides in a sterile gel formulation for intradermal application. This medical device PN HPT‐based is indicated for the prevention and correction of superficial signs of aging, for its moisturizing effect in thin or dry skin, and as a preparatory treatment before intensive aesthetic procedures, such as laser resurfacing or chemical peels [9].

PN HPT may be administered via a thin needle (usually 30–32 G) by injecting the solution into the dermis. Thanks to its versatility and manageability, it can be used in any skin area, including the scalp, forehead, eyebrows, cheekbones, eye contour, face, neck, abdomen, and buttocks. Depending on the clinician's assessment, different injection techniques may be used [10].

Given the increasing clinical use of polynucleotide‐based injectables and their favorable profile, generating real‐world evidence (RWE) is essential to better characterize their effectiveness and safety in routine practice [11]. Understanding how these treatments perform in everyday clinical practice may help guide evidence‐based decision‐making, optimize patient outcomes, and better align treatment expectations with actual patient experiences [11].

Our real‐world observational clinical data collection assessed the performance and safety of PN HPT in three frequently targeted anatomical areas: the face, neck, and décolleté. A dual assessment approach was employed, integrating physician evaluations with patient‐reported feedback and utilizing validated outcome measures, including the Global Clinical Improvement Scale (GCI‐S) and Global Aesthetic Improvement Scale (GAIS). Adverse events (AEs) were also monitored to assess tolerability, and patient satisfaction was evaluated to contextualize outcomes within the real‐life experience of individuals undergoing treatment.

## 2 Matherials and Methods

### 2.1 Study Design

We collected observational clinical data on the real‐world application of PN HPT within a post‐market clinical follow‐up (PMCF) framework. A survey utilizing both clinician and patient‐reported questionnaires was conducted to confirm the safety and performance of intradermal administration of PN HPT in the face, neck, and décolleté.

The clinical data collection included 106 questionnaires of adult patients who received intradermal infiltration with PN HPT aged 18 years and older, with no gender restrictions. However, the total number of patients was lower than the number of questionnaires collected, as individual participants could undergo treatment in multiple anatomical regions with a separate questionnaire completed for each treated area. Individuals with known hypersensitivity or prior allergic reactions to any injectable device component were excluded. Additionally, exclusions applied to those who were pregnant, breastfeeding, or had autoimmune diseases, present infections, dermatitis, inflammatory conditions in the treatment area, or any other condition considered inappropriate based on the clinician's assessment.

The product used in this clinical data collection, Plinest, is a class III CE‐marked medical device consisting of a single‐use, viscoelastic, and sterile gel containing PN HPT (40 mg/2 mL) intended for intradermal use. It was supplied in a 2 mL pre‐filled and pre‐assembled syringe [8].

The treatment protocol consisted of three sessions of intradermal injections in the specified body: the initial session at T0 (baseline and first treatment session), at T1 (second treatment session 2 or 3 weeks after T0) and at T2 (third and final session 2 or 3 weeks after T1). The performance of PN HPT was evaluated 3 months after the final injection, and safety measures were monitored throughout the clinical data collection period.

### 2.2 Performance Assessment

The main goal of this clinical data collection was to evaluate the rate of visible skin improvement, which was assessed using clinical evaluations and patient self‐reports. GCI‐S was the primary measurement tool. This valid assessment scale requires clinicians to determine how much the patient's situation has improved or worsened relative to a baseline state. It involves clinician assessments using a five‐point system: more than 75% improvement indicates an excellent result, 51%–75% shows marked improvement, 26%–50% shows moderate improvement, 0%–25% indicates slight improvement, and “N/A” indicates worsening [12].

The secondary objective was to evaluate global aesthetic improvement compared to baseline, as assessed by both clinicians and patients using the GAIS [13]. This five‐point scale evaluates the global aesthetic enhancement of the skin appearance. The rating categories included very much improved, much improved, slightly improved, no change, and worse. Additionally, the participants' overall experiences and willingness to undergo the procedure were assessed using a Likert scale [14].

### 2.3 Safety Assessment

A questionnaire was used to assess the safety of AEs, which were spontaneously reported by patients following intradermal infiltration.

### 2.4 Statistical Analysis

A descriptive analysis was conducted to summarize the patient characteristics, including age (mean ± standard deviation), Fitzpatrick skin type [15], and smoking habits. Variations among patients were assessed using a qualitative approach. This involves determining the percentage of patients who report an improvement of at least 1% compared to their pretreatment status, as evaluated by both the patients and clinicians.

## 3 Results

### 3.1 Demographic and Baseline Characteristics

Based on the analysis of 106 patient questionnaires completed by individuals who independently requested intradermal treatment, procedures were carried out in three anatomical regions: face (47 patients, 31%), neck (33 patients, 29%), and décolleté (26 patients, 26.5%). The cohort of 66 patients was predominantly female, with an average age of 49.5 years (±12.3). Participants' Fitzpatrick skin types ranged from I to IV. The demographic and baseline characteristics are detailed in Table 1. At the conclusion of the observational clinical data collection, 106 questionnaires were analyzed, reflecting outcomes from three treatment cycles per anatomical region, resulting in a total of 318 infiltrations.

**TABLE 1:** Demographic data and Fitzpatrick scale.

Face		
Age	Mean (SD) whole population	48.6 ± 12.1
	Range (years)	28–74
Sex (*N*)	Female	47
	Male	//
Smoker	Number	11
Fitzpatrick	Number	
1	2	
2	18	
3	22	
4	4	
Neck		
Age	Mean (SD) whole population	51.2 ± 12.5
	Range (years)	32–74
Sex (*N*)	Female	32
	Male	1
Smoker	Number	10
Fitzpatrick	Number	
1	2	
2	12	
3	14	
4	3	
Décolleté		
Age	Mean (SD) whole population	49.2 ± 12.3
	Range (years)	28–71
Sex (*N*)	Female	26
	Male	//
Smoker	Number	8
Fitzpatrick	Number	
1	2	
2	9	
3	14	
4	1	

The principal motivations for patients to seek treatment were compiled from a total of 59 responses, and analogous responses were categorized together. The corresponding frequency percentages are presented in Table 2.

**TABLE 2:** Reported reason for achieving treatment.

Reason for treatment	% of total responses (*n* = 59)
Aging (chronological/photoaging)	41
Skin roughness	30
Elastosis	11
Dryness associated with roughness	10
Skin laxity	5
Aging (reported separately)	3

### 3.2 Clinical Outcomes for Face Area

**FIGURE 1:**
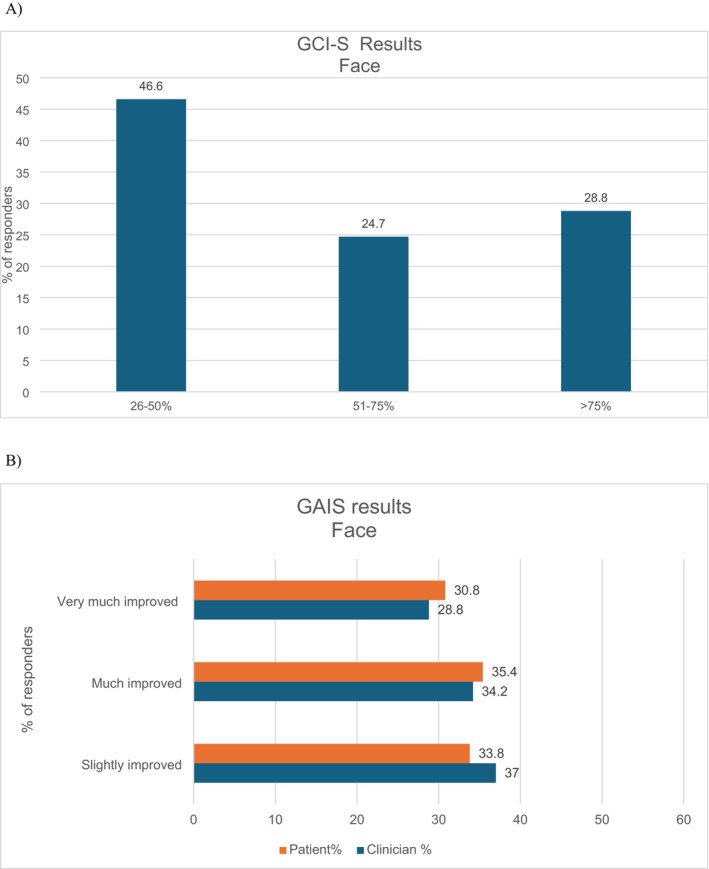
Evaluation of the treatment at the 3 months follow‐up for the face area. GCI‐S score provided by the clinician (A) and GAIS score provided by clinicians and patients (B). GAIS, Global Aesthetic Improvement Scale; GCI‐S, Global Clinical Improvement Scale.

At the three‐month evaluation (T2) rated by clinicians, 53.5% noted improvements in facial skin quality that ranged from “marked improvement” to “excellent improvement.” The remaining 46.6% reported “moderate improvement,” resulting in 100% of clinicians observing visible enhancements after PN HPT treatment (Figure 1A).

Patient and clinician assessments indicated high satisfaction rates and significant perceived improvements in the treated facial area. Specifically, 30.8% of patients reported being “very much improved,” which is moderately higher than the 28.8% reported by clinicians. Furthermore, 35.4% of patients considered their facial condition to be “much improved,” which is closely aligned with the 34.2% reported by clinicians. The “slightly improved” category was noted by 33.8% of patients, compared to a marginally higher proportion of 37% reported by clinicians (Figure 1B). These findings suggest a consistently high level of satisfaction and perceived effectiveness in both groups, with patients generally perceiving a somewhat more remarkable overall improvement.

### 3.3 Clinical Outcomes for the Neck Area

**FIGURE 2:**
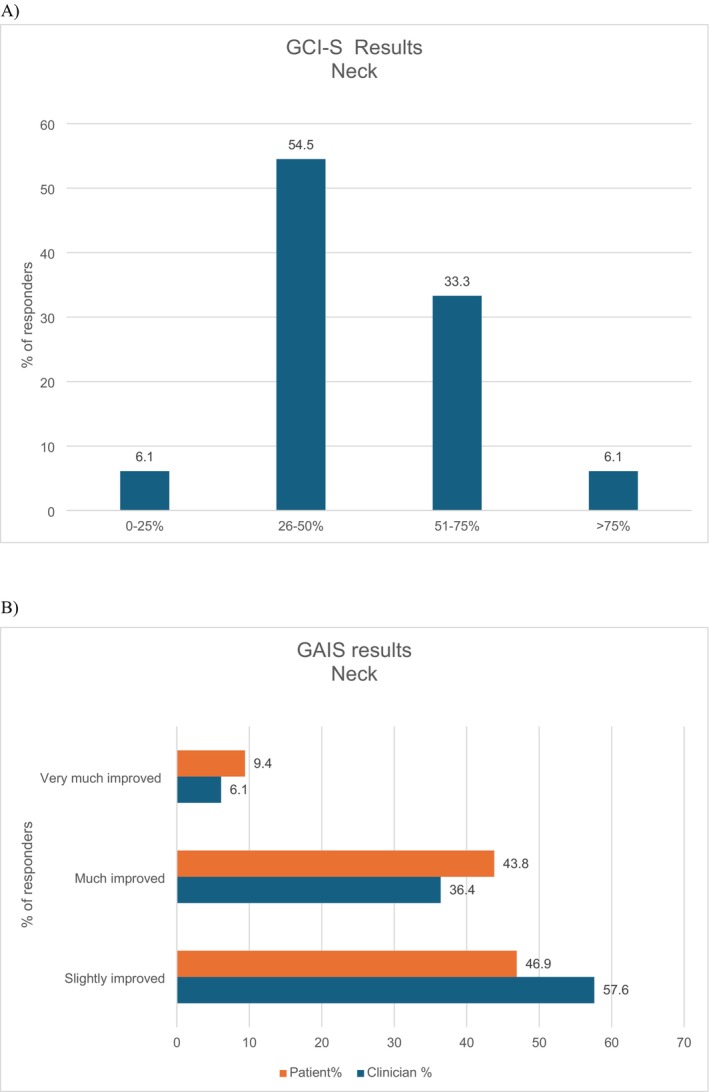
Evaluation of the treatment at the 3 months follow‐up for the neck area. GCI‐S score provided by the clinician (A) and GAIS score provided by clinicians and patients (B). GAIS, Global Aesthetic Improvement Scale.

Based on clinical evaluation, 87.8% of neck treatments were assessed as demonstrating “moderate and marked improvements,” while 6.1% of cases were deemed to exhibit excellent results. Conversely, in an equivalent percentage of cases, the outcomes were evaluated as showing no change or only a slight improvement (Figure 2A). Data from GAIS indicated substantial agreement between patient and clinician assessments. 53.2% of the patients rated their condition as either “very much improved” or “much improved,” whereas clinicians showed a lower percentage at 42.7%. Conversely, clinicians observed an improvement in nearly 58% of cases compared to almost 47% of patients. (Figure 2B).

### 3.4 Clinical Outcomes for the Décolleté Area

**FIGURE 3:**
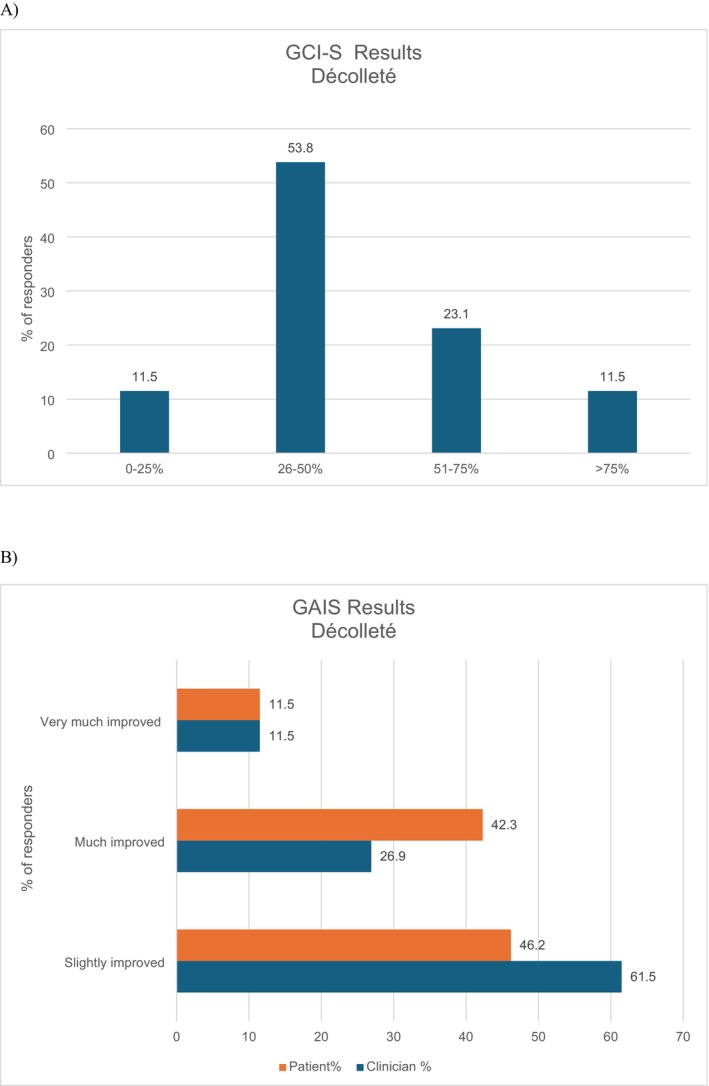
Evaluation of the treatment at the 3‐month follow‐up for the décolleté area. GCI‐S score provided by the clinician (A) and GAIS score provided by clinicians and patients (B). GAIS, Global Aesthetic Improvement Scale; GCI‐S, Global Clinical Improvement Scale.

Regarding the décolleté area, the GCI‐S scale indicated that clinicians reported moderate improvement in over half of the cases (53.4%). Furthermore, in more than 34% of the cases, the assessment was elevated to “very improved” or “very much improved”. In only 11.5% of cases, the clinicians observed no change, with only minimal changes recorded (Figure 3A). The assessments of clinicians and patients demonstrated a complete alignment in the “very much improved” category, with both groups reporting 11.5%. However, patients reported a “much improved” rating more frequently than clinicians (42.3% vs. 26.9%). Conversely, clinicians recorded a higher incidence of “slightly improved” ratings compared to patients (61.5% vs. 46.2%, respectively) (Figure 3B).

### 3.5 Patients' Satisfaction

Patients expressed high satisfaction with the treatment outcomes, as measured by the Likert scale. Most respondents indicated willingness to undergo the procedure again and reported overall satisfaction following treatment (Table 3).

**TABLE 3:** Likert scale of patients' satisfaction about the treatment.

Treated area	Patients open to repeat the procedure (%)	Patients reporting overall general satisfaction (%)
Face	97	97
Neck	96	100
Décolleté	97	100

### 3.6 Safety

The treatment was well‐tolerated across all anatomical districts. Most side effects were transient and classified as light in severity. No unknown or unexpected AEs were reported. Throughout all sessions, the most reported side effects were associated with the intradermal infiltration technique rather than the specific device. These included swelling, erythema, pain, bruising, and sensations of heat or itching. All side effects resolved spontaneously, with occasional use of bromelain tablets prescribed for swelling management in selected cases.

## 4 Discussion

Preventing skin aging and maintaining a healthy, relaxed appearance over time are among the key goals of preventive medicine, to which aesthetic medicine can significantly contribute [2]. Dermal fillers have gained great popularity over the years as they can be used to address many concerns related among others, to skin depressions, dull and lax skin, face remodeling and signs of skin aging. Dermal fillers also offer a minimally invasive alternative to facelift [16]. In this context, there is a growing interest in minimally invasive, well‐tolerated treatments with quick recovery times that enable patients to return to their normal daily activities immediately [2, 16]. This is also why long‐chain polynucleotides have become one of the most widely used procedures in aesthetic medicine [9].

Recent studies have confirmed PN HPT efficacy and tolerability in treating photo‐ and chrono‐aging of the skin, both on the face and in other areas of the body [17, 18, 19]. PN HPT acts as temporary fillers owing to the viscoelasticity of DNA fragments while promoting tissue repair and regeneration processes. As a result of these characteristics, the outcome is deeper and more natural rejuvenation. Data indicate that the moisturizing properties of PN HPT are superior to those of other dermal active ingredients, such as hyaluronic acid, thus providing a solid rationale to consider them as true “bioreactivating primers” of the skin [17, 18, 19]. The recent PN HPT Priming Board consensus document [6] describes the rationale for using PN HPT as a preparatory treatment to improve dermal tissue reactivity. Pretreatment with PN HPT may improve clinical outcomes and reduce AEs when combined with techniques such as CO_2_ laser, RF, microneedling, peeling, and fillers. Further support for the clinical utility of PN HPT comes from studies focused on specific dermatological conditions. PN HPT significantly improved moderate to severe atrophic acne scars in adult women compared to placebo. The effects were visible as early as 1 month after treatment and were maintained for 3 months, confirming the potential of PN HPT to support dermal remodeling even in structurally compromised skin [7].

Another randomized, prospective clinical trial demonstrates the effectiveness of intradermal PN HPT as a standalone treatment for rapidly enhancing dermal quality in moderate to severe nasolabial folds, with consistently reported aesthetic improvements by patients [5].

Medical devices utilizing PN HPT represent a safe and efficacious approach to aesthetic treatment, specifically targeting the rejuvenation, revitalization, and toning of facial and body skin [17]. Skin preparation and revitalization are conducted with the highest patient comfort, and any adverse effects observed are typically minor and transient [17].

Plinest, the class III medical device used in this clinical data collection, can be employed both as a single anti‐aging therapy to improve skin quality and as a preparatory treatment for other medical and surgical procedures (such as radiofrequency and surgery) [17]. In fact, the increase in collagen and non‐collagen substances in the dermis provides better conditions in the results obtained with other techniques for better responses and reduction in side effects [17]. Plinest supports and facilitates the natural increase in collagen and non‐collagen substances in the dermis providing better conditions in the results obtained with other techniques for better responses and reduction in side effects. The treatment protocols are particularly suitable for cases of light and moderate aging and photoaging, for thin and dry skin, to prevent skin aging and sun damage, and to prepare the skin before exposure to the sun [17].

Our results are in line with recent publication that an eight‐week intradermal treatment with a PN HPT based medical device produced clinically significant improvements in skin surface quality, texture, pigmentation, and brightness, with no AEs and patient‐reported benefits lasting up to 6 months [20].

Recent developments in aesthetic medicine have revealed that patients frequently assess their outcomes more favorably than clinicians. This divergence is primarily attributed to the distinct domains prioritized by each group during evaluation. According to a global perception survey conducted by Fabi et al., patients prioritize attributes such as skin quality, a natural appearance, and enhancements in specific areas like the under‐eye region. In contrast, physicians tend to emphasize symmetry, structural correction, and volumetric balance. This divergence in aesthetic objectives may elucidate why patients often perceive more pronounced benefits than clinicians [21].

Across all treatment areas, patients consistently rated their outcomes even more favorably than physicians, even though clinician assessments were already highly positive. This trend was particularly pronounced in the neck and décolleté areas, where patient‐reported improvements exceeded those noted by physicians. Among clinicians, the highest percentage of patients achieving excellent results was observed in the facial area, compared to those treated with intradermal injections in the neck and décolleté. These findings are consistent with existing literature, which highlights the subjective nature of aesthetic outcomes and the critical role of patient‐centered evaluations in cosmetic procedures [22].

Furthermore, the highest patient satisfaction rates were observed in the décolleté and neck areas, where 100% of respondents reported overall satisfaction and 96%–97% expressed willingness to repeat treatment. These findings are particularly relevant given the anatomical and structural complexity of these regions, which are often considered more challenging to treat using conventional rejuvenation techniques [23].

Additionally, data collected through the questionnaires highlighted that the treatment primarily addressed visible signs of skin aging, particularly those related to chronological and photoinduced aging. From a safety perspective, Plinest demonstrated a favorable profile with no serious or unexpected AEs recorded. These results are consistent with the properties of PN HPT injectables, that improve skin regeneration, collagen production, hydration, and elasticity, thanks to its moisturizing properties [24].

Safety data provided by this study confirmed the safety profile of Plinest build up during the 20 years presence on the markets. This research recognizes certain limitations. Firstly, the sample size was comparatively small, potentially reducing the generalizability of the results. Secondly, the follow‐up duration was limited, restricting understanding of the long‐term sustainability of clinical benefits. While the current clinical data collection concentrated on a 3‐month evaluation period, an extended follow‐up phase is underway to evaluate the durability of the clinical outcomes over time. Furthermore, previous studies have documented sustained improvements in skin quality with PN HPT treatment up to 6 months post‐injection, thereby supporting the long‐term benefits of this therapeutic approach [4, 20]. Future studies with longer follow‐up periods and a more diverse participant pool may be required to confirm these results.

However, by providing observational data from routine clinical practice, this clinical data collection aims to contribute to the expanding body of literature on PN HPT‐based aesthetic therapies and to support informed clinical decision‐making regarding their application in personalized and minimally invasive skin rejuvenation strategies.

## Author Contributions

All authors contributed to the study conception, data collection, analysis, and manuscript preparation. All authors reviewed and approved the final version of the manuscript.

## Ethics Statement

This study was conducted in accordance with the principles of the Declaration of Helsinki. As a non‐interventional, observational data collection on CE‐marked medical devices, no formal approval from an ethics committee was required under local regulations. All patients provided written informed consent before undergoing treatment.

## Consent

No identifiable images of patients are included in this article.

## Conflicts of Interest

The authors declare no conflicts of interest.

## Data Availability

The data that support the findings of this study are available on request from the corresponding author. The data are not publicly available due to privacy or ethical restrictions.
